# The Value of and Indications for Incision of Ear Drum in Otitis Media*Revision of a paper read before the Canal Zone Medical Association, October 13th, 1909.

**Published:** 1913-06

**Authors:** Richard M. Nelson

**Affiliations:** Atlanta, Ga.


### THE VALUE OF AND INDICATIONS FOR INCISION OF EAR DRUM IN OTITIS MEDIA.*

By Richard M. Nelson, M. D., Atlanta, Ga.

Fridenberg of New York says (1): "As a matter of fact, acute otitis media is to this day considered a self-limited disease, with a typical cyclical course and critical defervescences, like pneumonia. Zaufal and his school, in common with other German otologists, have statistics to show, or more properly to make

*Revision of a paper read before the Canal Zone Medical Association, October 13th, 1909.

it appear, that spontaneous perforation is preferable to that made by the knife; that paracentesis is generally unnecessary, and that the results of the expectant treatment are at least equal to those obtained by early surgical interference. The fallacy and folly of these views need not be pointed out to American otologists, nor need they be reminded that the conscientious surgeon will not be consoled for the loss of a single life through needless waiting, when a simple, safe and rapid procedure would have averted death." In his article, Dr. Fridenberg lays great stress upon his method of aspiration after paracentesis ha sbeen performed, and he appears to consider it as important as the paracentesis itself. In only one in the series of 105 cases of otitis media upon which the conclusion arrived at in this paper are based, was aspiration practiced, and as that case disappeared from view before any improvement in the ear condition was noticeable, I cannot say that that method was given a trial worth basing any conclusions upon. It may be that had I aspirated pus from the middle ear through the paracentesis opening in all my cases, the results would have been even better than they are.

I think it well just here to take exception to the term "paracentesis," and to substitute, as suggested by Dench (2) of New York, the word "incise." We are so apt to associate in our minds the word "paracentesis" with a small opening made to tap a body cavity. Whereas in opening the ear drum for drainage of the middle ear it is of the highest importance not only to incise the drum, but to incise it freely. As to the danger of the opening in the drum remaining for any length of time and not healing perfectly, the danger is really the other way, the wound being liable to heal too quickly, often in as short a time as 24 to 36 hours. Probably the best rule to follow as to the location of the incision is to incise at the point of bulging, if the bulging is localized in some one portion of the ear drum. And, where the bulging is general, to select the posterior inferior quadrant of the membrana tympani as the safest and best place to incise. In this location an incision can be made from the lower border of the membrane upward and backward to the supero-posterior border, with the point of the knife plunged deeply enough to incise freely, not only the drum membrane, but also the mucosa covering the inner wall of the middle ear, without the least danger of injuring the ossicular chain or other delicate structures of the ear. Many make the cut in this way believing the incision through the congested mucosa, and free bleeding from same, aid very materially in relieving both objective and subjective symptoms. In my hands the instrument that has proven the simplest and best for making the incision is the Von Graafe cataract knife. Partial anesthesia is obtained, and the nearest approach to asepsis or antisepsis arise in the following way· A strip of fine gauze packing soaking wet with 10% phenolized glycerin is gently inserted through the ear speculum so that it lies snugly against the drum. The speculum is removed, and the rest of the packing inserted so as to fill the entire external auditory canal. After thirty minutes the packing is removed, and a cylinder of cotton soaked with 10% Cocaine hydrochlor, and warmed, is introduced in the same way and left in position about ten minutes, and then removed and the ear again tamponed with warm phenolized glycerin as at first, but this time for only about five or ten minutes. The drum can now be incised with as little pain as can be prevented by any method of local anesthesia, and with a very near approach to surgical asepsis. I have found phenolized glycerin used in this way, not only more efficacious as an antiseptic, but more pleasant to the patient through the anesthetic or analgesic effect of the phenol, than where peroxide or alcohol used to render the field for this little operation surgically clean· To obtain material for bacteriological examination, from the middle ear, without contamination from ear canal, the latter method is probably preferable. Incision is at times of value not only from the standpoint of preventing the symptoms to be enumerated presently, but also as a diagnostic measure. On the whole, the bacteriology of otitis media is unfortunately of far more scientific than practical interest or value.

The bacteria most frequently found in acute otitis media are: Diplococcus pneumoniae, streptococcus pyogenes, staphylococcus aureous and albus, their frequency being in the order named. Among other bacteria more or less frequently found are Bacillus pneumoniae, B. diphtheriae, B. tuberculosis, B. influenzae, B. pyogenes foetidus and B. coli communis. (3), (4). In chronic suppurative otitis media a bacterium is seldom found in pure culture. The infection is usually due to mixed organisms, the predominant one being a staphylococcus. The organism that caused the acute condition usually becoming either attenuated or is replaced by others. (3), (4).

I will present briefly the results obtained at Colon Hospital, Cristobal, Canal Zone, between November, 1906, and March, 1909, in the cases of otitis media in which it was found necessary to incise the ear drum. There were many cases in which the operation was not done, as sufficient drainage was secured through the spontaneous perforation of the drum membrane, or whose symptoms had not been such as to necessitate incision. These cases will be left out of the discussion and consideration will be given only to the small proportion of the total number of patients in which the membrana tympani was incised. Between the dates mentioned I incised the drum in 102 cases of acute and in 3 cases of chronic otitis media. Among the cases of acute otitis media in which the drum was incised, only two subsequently required mastoid operations.

I did a mastoid operation upon one man with acute suppurative otitis media and acute mastoiditis due to the streptococcus, as verified by the bacteriological laboratory, in whom not a trace of the drum membrane could be found at the first examination. The ear drum having been entirely destroyed during the fifteen days that ear symptoms existed before his admission to my service at the hospital. Had I seen this patient earlier in the disease, however, I would have done the mastoid operation as soon as streptococcus mastoiditis diagnosed after paracentesis membrana tympani, without waiting an hour longer than absolutely necessary. Delay being so dangerous in these cases, and very little to be expected from a mere incision of the ear drum.

Barnhill, of Indianapolis, states (4), that "Acute Mastoiditis is in nearly every instance secondary to a previous infection and suppuration of the tympanic cavity. It has been estimated that not over 1% of all cases of this disease originates in the antrum itself, and of the small number nearly all cases of primary mastoiditis are due to external trauma." I had one such case in which extension occurred from an abscess over the mastoid to the mastoid cells and antrum. Neither before nor after mastoid operation was there the slightest objective or subjective symptom of otitis media. Paracentesis membrana tympani was done on first examination for diagnostic purposes purely, and with entirely negative results. This case is mentioned merely to emphasize the rarity of mastoiditis without otitis media·

I wish to lay special emphasis upon the fact that the 102 cases of acute otitis media in which the ear drums were incised, all showed objective, as well as subjective, symptoms of the beginning extension into the mastoid antrum, if not into the mastoid cells, as follows: Tinnitus aurium. bulging, reddened or whitened drum membrane, with obliteration of its landmarks; tenderness of mastoid at either antrum or tip or both, an evening rise of temperature from 99 to 100, or 102 or 103 degrees Fahrenheit. In mose cases there was a leucocytosis of 10,000, 12.-000, or 15,000. In the differential white count there was generally an increase in the polymorphonuclears to 70, 75 or 80%. In practically every case, including the two in which mastoid operations were found necessary later, all the symptoms enumerated above disappeared, either wholly or in part, within 24 hours after incision. Pain was relieved and tenderness diminished usually within 24 hours, and the temperature generally dropped to normal within this period, and the leucocytosis lessened· Throbbing pain was relieved almost without exception, within a few minutes after incising the drum. The sharp pain caused by this incising of the sensitive ear drum wore off in 5 or 10 minutes. I have seen the statement made in an article in some medical journal that solutions of cocaine and adrenalin instilled in the ear canal will render paracentesis entirely painless. In a rather extensive experience before and after the dates mentioned earlier in this paper, as well as during that period, I have seen only one or two cases where paracentesis was done that did not suffer more or less sharp, if momentary, pain. And very probably these patients suffered pain, but felt ashamed to admit that they did. I feel confident that practically all otologists with an experience equaling, or more extensive than mine, will agree with the statement that there is absolutely no reliable method for rendering incision of the ear drum painless short of giving a general anesthesia. General anesthesia is advisable, if not absolutely necessary, in children, and in highly nervous adults. The simple Schwartze mastoid operation was done on the two patients whose mastoid symptoms did not subside after incising the drum during acute otitis media, and it is noteworthy that both patients made uneventful recoveries, and both had practically normal ear drums and unimpaired hearing in both ears when discharged from treatment. These two cases, as well as many of the others with evidence of beginning invasion of the mastoid, showed the bulging of the posterior-superior canal wall adjacent to the drum, so often considered as pathognomonic of mastoiditis. This was found in many cases but by no means in all.

In those cases of chronic suppurative otitis media in which the drum was incised, only temporary amelioration of the ear condition was obtained, and the procedure had to be repeated several times in all of them.

Chronic Case No. 1.—In this case paracentesis was done 18 times, and also a simple Schwartze, and later an ossiculectomy without success as far as lessening the purulent otorrhea was concerned. Finally the radical mastoid operation was resorted to, and after a rather prolonged after-treatment, including curetting several times the eustachian opening in the middle ear from which pus came for some time, all symptoms ceased.

Chronic Case No. 2.—Purulent otorrhea for 18 years, since he was 3 years old. Moderate sized perforation ear drum of the affected ear, the left. Chiefly complained of an inability to either whistle or shut his left eye for one week. Typical facial paralysis on this side. Facial paralysis diagnosed as being due to chronic mastoiditis, either directly from pressure upon facial nerve in its course under the facial ridge between middle ear and antrum, or indirectly as it were, from absorption of pus or toxins in this location. On opening the mastoid, it was found of such ivory-like hardness, from chronic mastoiditis, and landmarks and pneumatic cells so completely obliterated, that further exploration by this route was abandoned, and an immediate ossiculectomy performed. Facial paralysis gradually disappeared and the otorrhea diminished, until when patient was discharged a month later there were no symptoms of the paralysis left, and only a slight discharge of pus from the ear.

Chronic Case No· 3.—Case apparently rather hopeless from time first seen. He was transferred from the medical to the surgical wards with a provisional diagnosis of brain abscess. As this case appears to me to be of particular interest from several standpoints, I will give a rather detailed account of it.

May 3rd, 1907, Manuel M., Spanish-American. As patient nearly comatose no history obtainable· Chart showed subnormal pulse and temperature, the latter 97 degrees and lower, and the former 30 to 40 a minute. Examination eyes. —Conjunctival hyperemia convergent strabismus, well marked lateral nystagmus. Typical choked disc found on opthalmoscopic examination after pupils dilated with atropine. Choked disc measured about 3 diopters one eye, apparently 2 or 2 1-2 the other, but nystagmus rendered accurate measurement difficult. Examination ears—Right ear drum whitish, dull, lacking luster and bulging outward. On incision a thick, yellowish pus of foul odor was evacuated. No edema mastoid soft tissues. As to tenderness, patient in such a stupor as to be apparently insensible to pain. Left ear showed some catarrhal otitis media only, so drum not incised. May 6th—Mastoid cells opened, pus and necrotic bone removed. Further operative exploration revealed an abscess of cerebellum, an ounce or more of thick yellow pus being evacuated. After operation, patient never roused from stupor. The temperature rose gradually until it reached 106 degrees just before death occurred. Recovery from cerebellar abscess is rare. On autopsy the path the infection had traveled could be clearly traced from the middle ear, through the internal ear, internal auditory meatus, and, finally, to the cerebellum. Of brain abscesses no less than 30%, according to McKernon of New York (5), are caused by a suppurative otitis media.

Now, if a suppurative process in the middle ear were always, or nearly always, strictly confined to this cavity, it might with justice be considered the trivial affair that so many patients and medical men appear to regard it. But, unfortunately, it is a condition fraught with great possibilities of danger from extension of the infection to neighboring and important structures. Extension into the mastoid antrum and into the mastoid cells may very frequently occur, and from these cavities extension may occur, resulting in thrombosis of the lateral sinus, extra-dural abscess, cerebral and cerebellar abscesses, and meningitis. The suppurative process may also penetrate the inner wall of the tympanum, causing suppurative labyrinthitis, with possibly a subsequent invasion of the brain through the internal auditory meatus. The case I have just described in such detail was undoubtedly one of this kind.

All the chronic cases referred to above were chronic already when first seen at the clinic. Noting the results obtained in the acute cases studied in this paper, is it not reasonable to suppose that by early incision of the drum, or other appropriate treatment, such serious results of the disease would have probably been averted. These cases might even have been prevented from ever having become chronic, and even useful hearing preserved for them.

My clinic at Colon Hospital grew enormously between March, 1909, and August, 1912, the date I left Panama. The clinic in 1909 averaged about 300 a month. The Out-Eye, Ear, Nose, and Throat Clinic, during my last year at Colon Hospital, ranged between 1,000 and 1,200 a month, exclusive of hospital ward patients. My vastly increased opportunity for clinical study, during my last years in Panama, and the greatly widened experience incidental thereto, have only served to confirm still further the conclusions forced upon me by my study of otitis media undertaken in 1909 from the limited data then in my possession.

### BIBLIOGRAPHY.

(1) Fridenberg, Dr. Percy, Journal American Medical Association, Jan. 4th, 1908.

(2) Dench, in Section of "Surgery of the Ear," in "Keene's Surgery."

(3) Reik, Dr. H., "The Pathology of Middle Ear Suppuration," Journal A. M. A., August, 1907.

(4) Barnhill and Wales, Text book, "Modern Otology," Edit. 1908.

(5) McKernon, Journal A. M. A., December, 1909.

805-807 Empire-Life Building.

---

### SURGICAL SUGGESTIONS.

The perineal route is to be preferred for the removal of the cancerous prostate.

Renal calculi occasionally cause constant dull pain without any "attacks."

The lining membrane of a maxillary bone cyst is usually easily removable complete with forceps.

Midline sinuses or swellings are always suggestive of dermoid cysts.

A suppurating perineal cyst may discharge through the scrotum or at the peno-scrotal angle, suggesting a peri-urethral abscess.

Beck's bismuth paste will sometimes cure a persistent narrow empyema sinus; but it cannot cure a persistent empyema cavity, and in such cases it is very apt to cause serious or fatal bismuth poisoning.

When bismuth paste is used in any considerable quantity (an ounce or more) for x-ray diagnosis it should be promptly washed out of the wound with olive oil, to avoid poisoning.—American Journal of Surgery.

